# Uncoupling protein-2 attenuates palmitoleate protection against the cytotoxic production of mitochondrial reactive oxygen species in INS-1E insulinoma cells

**DOI:** 10.1016/j.redox.2014.11.009

**Published:** 2014-11-28

**Authors:** Jonathan Barlow, Verena Hirschberg Jensen, Charles Affourtit

**Affiliations:** a School of Biomedical and Healthcare Sciences, Plymouth University, Drake Circus, Plymouth PL4 8AA, UK

**Keywords:** Pancreatic beta cells, Glucolipotoxicity, Cytoprotection, Mitochondrial dysfunction, Uncoupling protein-2 (UCP2), Reactive oxygen species, INS-1E insulinoma cells, Non-esterified fatty acids, Obesity, Type 2 diabetes, BSA, bovine serum albumin, DAPI, 4′,6-diamidino-2-phenylindole, DHE, hydroethidine, DPBS, Dulbecco's modified PBS, EDTA, ethylenediaminetetraacetic acid, FBS, fetal bovine serum, GSIS, glucose-stimulated insulin secretion, Hepes, 4-(2-hydroxyethyl)-1-piperazineethanesulfonic acid, KRH, Krebs–Ringer–Hepes buffer, MitoSOX, DHE conjugated to triphenylphosphonium, NEFA, non-esterified fatty acid, PBS, phosphate-buffered saline, ROS, reactive oxygen species, SDS, sodium dodecyl sulphate, TBST, Tris-buffered saline with Tween-20, UCP2, uncoupling protein-2

## Abstract

High glucose and fatty acid levels impair pancreatic beta cell function. We have recently shown that palmitate-induced loss of INS-1E insulinoma cells is related to increased reactive oxygen species (ROS) production as both toxic effects are prevented by palmitoleate. Here we show that palmitate-induced ROS are mostly mitochondrial: oxidation of MitoSOX, a mitochondria-targeted superoxide probe, is increased by palmitate, whilst oxidation of the equivalent non-targeted probe is unaffected. Moreover, mitochondrial respiratory inhibition with antimycin A stimulates palmitate-induced MitoSOX oxidation. We also show that palmitate does not change the level of mitochondrial uncoupling protein-2 (UCP2) and that UCP2 knockdown does not affect palmitate-induced MitoSOX oxidation. Palmitoleate does not influence MitoSOX oxidation in INS-1E cells ±UCP2 and largely prevents the palmitate-induced effects. Importantly, UCP2 knockdown amplifies the preventive effect of palmitoleate on palmitate-induced ROS. Consistently, viability effects of palmitate and palmitoleate are similar between cells ±UCP2, but UCP2 knockdown significantly augments the palmitoleate protection against palmitate-induced cell loss at high glucose. We conclude that UCP2 neither mediates palmitate-induced mitochondrial ROS generation and the associated cell loss, nor protects against these deleterious effects. Instead, UCP2 dampens palmitoleate protection against palmitate toxicity.

## Highlights

• Palmitate exposure induces mitochondrial superoxide in INS-1E cells.
• Mitochondrial superoxide correlates inversely with INS-1E cell number.
• Palmitate does not change uncoupling protein-2 (UCP2) level in INS-1E cells.
• UCP2 does not affect palmitate-induced mitochondrial superoxide or INS-1E cell loss.
• UCP2 dampens palmitoleate protection against palmitate toxicity.

## Graphical abstract

**Figure:**
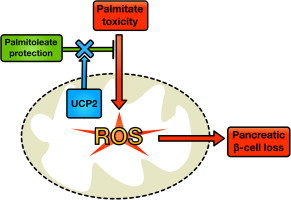

## Introduction

High levels of circulating glucose and non-esterified fatty acids (NEFAs) impair pancreatic beta cell function and thus contribute to the pathogenesis of Type 2 diabetes [1]. We have recently shown in INS-1E insulinoma cells that mitochondrial dysfunction is involved in this beta cell glucolipotoxicity [2]. Specifically, overnight palmitate exposure at high glucose causes oxidative phosphorylation defects that are related to impaired glucose-stimulated insulin secretion. Concomitantly, palmitate exposure leads to increased reactive oxygen species (ROS) that are associated with cell loss. In agreement with the notion that unsaturated NEFAs provide protection against the harmful effects of saturated NEFAs [3] the deleterious effects of palmitate on ROS and INS-1E cell viability are largely prevented by its monounsaturated counterpart palmitoleate [2]. Oxidative stress has been linked extensively to beta cell dysfunction and death that give rise to Type 2 diabetes [4–7]. Indeed, ROS scavenging has been reported to improve beta cell health under glucolipotoxic conditions [8,9]. Beta cells generate ROS in the cytoplasm through activity of a plasma-membrane-bound NADPH oxidase and in mitochondria as a consequence of nutrient catabolism [7]. The origin of the recently reported palmitate-induced ROS in INS-1E cells [2] is unclear at present as is the mechanism underlying the protective effect of palmitoleate.

ROS are non-canonical signals in glucose-stimulated insulin secretion (GSIS) [10,11] and studies with isolated islets [12] and INS-1E cells [13] have revealed that uncoupling protein-2 (UCP2) attenuates GSIS by dampening ROS production. This acute regulatory effect of UCP2 on beta cell function is consistent with the relatively strong GSIS exhibited by the first-established *Ucp2*-ablated mouse strain [14], which suggested a pathological role for UCP2 in the development of beta cell dysfunction and Type 2 diabetes [15]. Indeed, studies involving this original genetic knockout strain demonstrated that UCP2 restricts the insulin secretory capacity of mice fed a high fat diet [16] and that UCP2 mediates beta cell defects caused by free fatty acids [17]. However, work on more recently established *Ucp2*-deficient mouse strains [18] has suggested a physiological instead of a pathological role for UCP2 as the protein has been attributed a protective function in assisting beta cells to deal with sustained oxidative stress [6]. Such stress is for example encountered after chronic fatty acid exposure [19,20]. A protective physiological role for UCP2 is consistent with its reported ROS-dampening effect [13], but predicts that UCP2 activity would ameliorate harmful effects of free fatty acids and high fat diet rather than mediate them. Evidently, UCP2 involvement in beta cell glucolipotoxicity remains unclear [1].

With the current study we aimed (i) to establish in which cellular compartment ROS arise in INS-1E cells incubated under glucolipotoxic conditions and (ii) to clarify if and how UCP2 regulates ROS production in NEFA-exposed cells. We reveal that palmitate-induced ROS predominantly emerge from mitochondria and that these ROS correlate strongly with loss of cell viability. We show that neither palmitate nor palmitoleate significantly affect UCP2 protein level. Consistently, knockdown of UCP2 by RNA interference does not alter palmitate-induced mitochondrial ROS or associated cell loss. Interestingly, UCP2 knockdown amplifies the dampening effect of palmitoleate on palmitate-induced ROS and augments palmitoleate protection against palmitate-provoked cell loss at high glucose.

## Experimental

### Cell culture

INS-1E insulinoma cells were donated by Prof. Noel Morgan (University of Exeter Medical School, UK) and maintained in RPMI-1640 medium (pH 7.4) containing 11 mM glucose, 5% (v/v) fetal bovine serum (FBS), 10 mM Hepes, 1 mM sodium pyruvate, 50 U/mL penicillin, 50 µg/mL streptomycin, 500 µM β-mercaptoethanol and 2 mM glutaMAX (Catalogue #35050-061, Life Technologies). INS-1E cells were seeded at 60,000 cells/well and, at 70–80% confluence, exposed to NEFAs for 24 h in serum-free RPMI containing 11 or 4 mM glucose. NEFAs were administered in conjugation to bovine serum albumin (BSA) as described before [2] and control cells were exposed to BSA alone. For RNA interference experiments, INS-1E cells were seeded at 60,000 cells/well, grown overnight to 50–60% confluence and then transfected with 200 nM *Ucp2*-targeted or scrambled (silencer negative control # 1) siRNA oligonucleotides (both from Ambion, Huntingdon, UK) that were complexed with 1.67 µg/mL Lipofectamine 2000 (Invitrogen, Paisley, UK) in serum-free RPMI. After 24-h incubation with siRNA–lipofectamine complexes, growth medium was replaced with serum-free RPMI supplemented with either 11 or 4 mM glucose and with appropriate NEFA:BSA conjugations. Transfected cells were exposed to NEFAs for 24 h.

### ROS

Mitochondrial and cytoplasmic ROS levels were estimated from MitoSOX (Catalogue #M36008, Life Technologies) or DHE (Catalogue #D-1168, Life Technologies) oxidation rates respectively as described before [13]. Cells seeded, transfected and NEFA-exposed on 96-well plates were washed into glucose-free Krebs–Ringer–Hepes (KRH) medium comprising 135 mM NaCl, 3.6 mM KCl, 10 mM Hepes (pH 7.4), 0.5 mM MgCl_2_, 1.5 mM CaCl_2_, 0.5 mM NaH_2_PO_4_ and 2 mM l-glutamine, and incubated in this medium (±15 µM antimycin A) for 30 min in a 37 °C air incubator. Next, plates were transferred to a multimode plate reader (PHERAstar FS, BMG Labtech) and following injection of either 5 µM MitoSOX or 100 µM DHE, fluorescence was monitored at 28-s intervals for 30 min. Fluorescent MitoSOX and DHE oxidation products were excited at 510 nm and light emission was detected at 580 nm. The plate reader's focal height was set at 3.4 mm and its gain was fixed between different experiments.

### Cell number

**Fig. 1:**
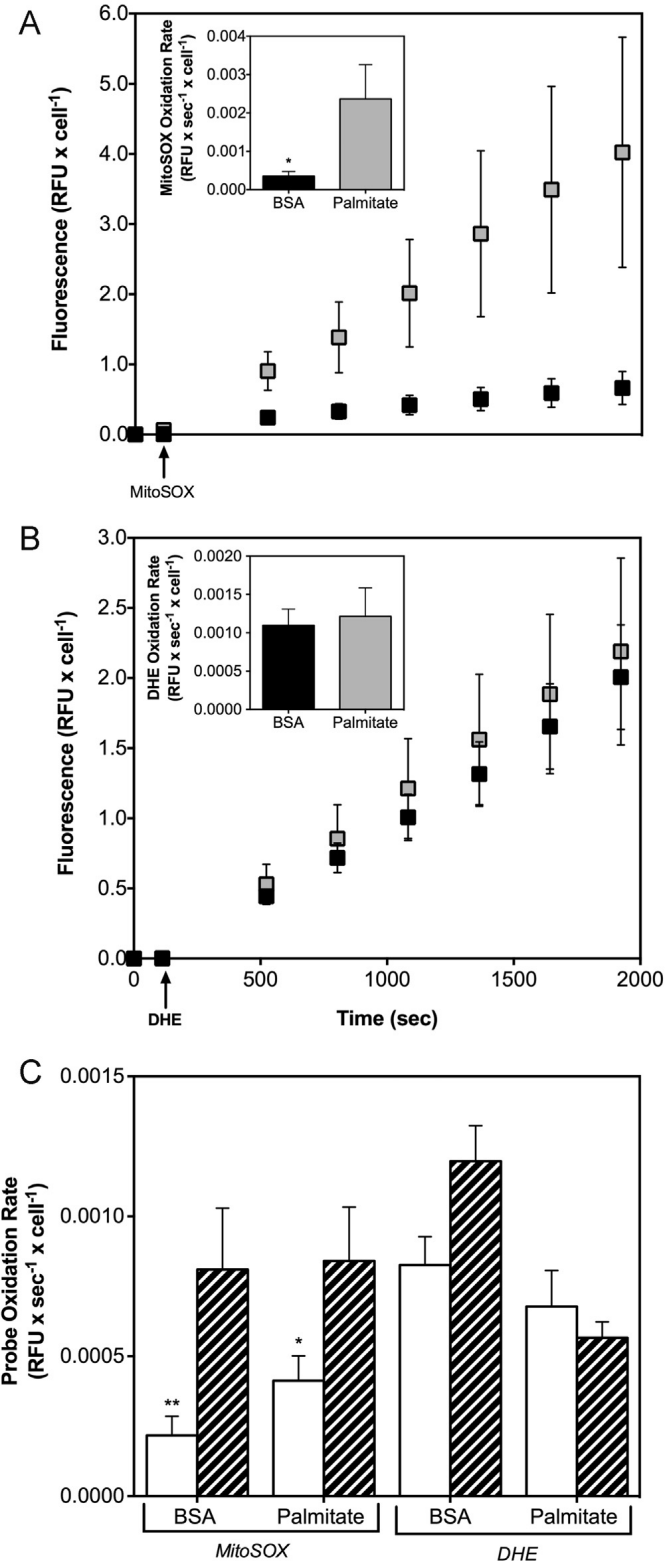
Palmitate-induced ROS are mitochondrial. Mitochondrial and cytoplasmic superoxide levels were estimated from MitoSOX (5 µM, panels A and C) and DHE (100 µM, panels B and C) oxidation rates, respectively, in INS-1E cells exposed for 24 h at 11 mM glucose to BSA-conjugated palmitate or BSA alone. (Panels A and B) Probes were injected at times indicated by the arrows and background-corrected fluorescence was recorded at 28-s intervals; for clarity, only a selection of measurements is shown. Relative fluorescence units (RFU) were normalised to cell number using mean INS-1E viability data (Fig. 5). Probe oxidation rates (inset panels) were calculated from the slopes of the progress curves; except for the first 4 measurements after probe addition, all data were included in these calculations. Black and grey symbols (both main and inset panels) reflect BSA control and palmitate-exposed cells, respectively. Data are mean±SEM from 3–4 independent exposures that involved 7–8 replicates per treatment. Statistical significance of rate differences was tested by unpaired Student's *t*-tests. * Differs from the equivalent palmitate condition (*P*<0.05). (Panel C) Probe oxidation rates were determined ±15 µM antimycin A (striped and white bars, respectively). Data are mean±SEM from 3–11 independent exposures that involved 7–8 replicates per treatment. Statistical significance of rate differences was tested by two-way ANOVA with Tukey's post-hoc analysis. ^⁎^, ^⁎⁎^ Differs from the equivalent antimycin A condition (*P*<0.05 and *P*<0.01, respectively).

Densities of attached INS-1E cells were determined by fluorescent DAPI-staining. Cells seeded, transfected and NEFA-exposed on 96-well plates were washed into 200 µL/well PBS, fixed with 4% (w/v) paraformaldehyde for 20 min at room temperature, and then incubated with DAPI (0.5 µg/mL in PBS) for another 15 min at room temperature. To minimise background fluorescence, excess DAPI was removed by washing 4× with PBS before measuring total-well fluorescence (*λ*_ex/em_=358/461 nm) using a multimode plate reader (PHERAstar FS, BMG Labtech) in bottom-reading and well-scanning mode. Standard curve-derived cell numbers were used to quantify NEFA effects on cell viability (Figs. 5 and 6) and to normalise ROS probe oxidation rates (Figs. 1, 3 and 4).

### UCP2 protein

**Fig. 2:**
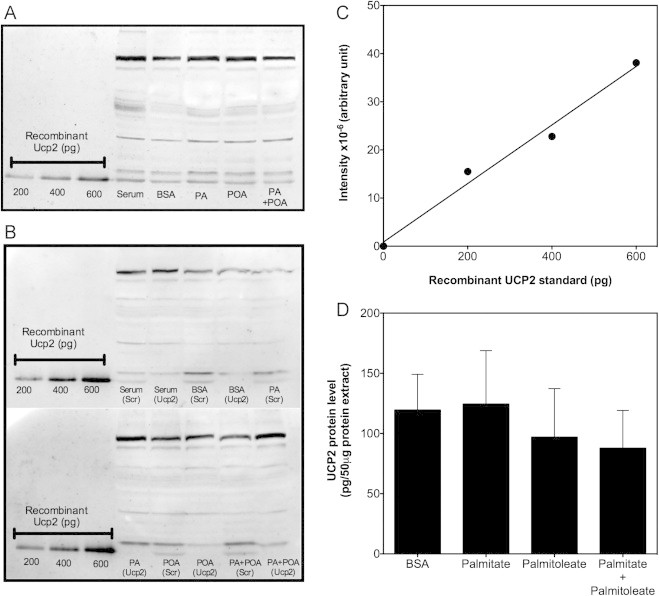
UCP2 protein in INS-1E cells is not affected by palmitate and/or palmitoleate. (Panel A) Typical Western blot showing cross-reactivity of UCP2 antibodies with partially purified recombinant human UCP2 (Recombinant Ucp2) and INS-1E proteins separated by SDS-PAGE (see Experimental section). Proteins were isolated from cells exposed for 24 h at 11 mM glucose to BSA-conjugated palmitate (PA) and/or palmitoleate (POA), BSA alone (BSA) or serum-supplemented growth medium (serum). (Panel B) Typical blots showing data from cells transfected with *Ucp*2-targeted or scrambled siRNA oligonucleotides (Ucp2 and Scr, respectively) before fatty acid exposure. (Panel C) Typical relation between signal intensity and amount of recombinant UCP2 as determined for each individual experiment (cf. panels A and B) to allow comparison of UCP2 levels between different samples. Membrane images were analysed with ImageQuant software using its *1D gel analysis* feature: background in defined lanes was subtracted by the *rolling ball* function, bands reflecting known hUCP2 amounts were boxed, and by applying the *quality calibration* function the presented relation was generated. (Panel D) UCP2 content approximated as picograms per 50 µg total extracted protein. Data represent mean±SEM from 3 independent fatty acid exposures. Data were analysed statistically by one-way ANOVA with Tukey’s post-hoc analysis revealing no significant differences between conditions.

INS-1E cells were seeded in 25-cm^2^ tissue culture flasks (BD Bioscience) at 1×10^6^ cells/flask and transfected with scrambled or *Ucp2*-targeted siRNA at 50–60% confluency. After 24-h incubation, cells were washed into serum-free RPMI and exposed to NEFA:BSA conjugations for 24 h. Cells were then removed using a cell scraper, washed with ice-cold DPBS and lysed in ice-cold buffer containing 50 mM Tris–HCl (pH 8.0), 1 % (v/v) Nonidet P40, 0.25 % (w/v) sodium deoxycholate, 0.1% (v/v) SDS, 150 mM NaCl, 1 mM EDTA and 500× diluted protease inhibitor (#P8340, Sigma-Aldrich). Following centrifugation of the cell lysates (14,000*g* for 20 min at 4 °C) protein content of the supernatants was estimated with a bicinchonic acid assay (Catalogue #23227, Thermo Scientific) and aliquots containing 50 µg protein were mixed with ice-cold acetone and left overnight at −20 °C. Subsequent centrifugation (10,000*g* for 15 min at 4 °C) yielded precipitated protein pellets that were solubilised and reduced in 20 µL buffer containing 10% (v/v) glycerol, 50 mM Tris–HCl (pH 6.8), 2% (v/v) SDS, 2% (v/v) β-mercaptoethanol, 0.1 mM EDTA and 0.01% (w/v) bromophenol blue. Proteins were separated by SDS-PAGE and transferred to nitrocellulose membrane (Whatman Protan, BA85) at room temperature using a semi-dry transfer cell (Trans-Blot SD, BIO-RAD) set at 20 V for 30 min. Rocked (65 rpm) at room temperature, membranes were blocked for 2 h in TBST [20 mM Tris–HCl (pH 7.5), 50 mM NaCl and 0.1% (v/v) Tween-20] containing 3% (w/v) skimmed milk powder (MARVEL). Primary UCP2 antibodies (sc-6525, Santa Cruz) were then added to the blocking buffer at 0.4 µg/mL. Following overnight incubation at 4 °C (rocked at 100 rpm), membranes were washed 4× over a period of 30 min with TBST, and then incubated for 2 h at room temperature with 0.2 µg/mL peroxidase-conjugated secondary antibodies. Following 4 TBST washes over a period of 30 min, antibody cross-reactivity was detected by chemiluminescence (ECL Prime, Amersham) using a LAS 4000 camera (GE Healthcare) collecting images at 30-s intervals for 5 min. Membrane images were analysed with ImageQuant TL version 7.0 (GE Healthcare). Band intensities were normalised to total protein per lane by routinely staining membranes with Pierce™ GelCode Blue reagent (Catalogue #24590, Thermo Scientific) after UCP2 detection. As described fully in Fig. 2, UCP2 protein levels were compared between samples by normalising to partially purified recombinant human UCP2 standards donated by Dr. Paul Crichton (MRC Mitochondrial Biology Unit, Cambridge, UK).

### Statistics

Statistical significance of mean differences was tested by Student’s *t*-test or ANOVA as specified in the figure legends using GraphPad Prism Version 6.0 for Mac OS X (GraphPad software, San Diego, CA, USA).

## Results

### Palmitate-induced ROS emerge from mitochondria

**Fig. 3:**
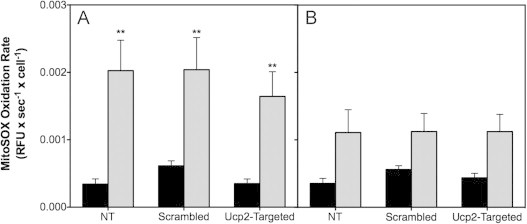
UCP2 knockdown does not change the effect of palmitate on mitochondrial ROS. MitoSOX oxidation rates were determined (see Fig. 1) in non-transfected INS-1E cells (NT) or cells transfected with scrambled or *Ucp2*-targeted siRNA oligonucleotides. Cells were exposed for 24 h at 11 or 4 mM glucose (panels A and B, respectively) to BSA-conjugated palmitate or BSA alone (grey and black bars, respectively). Data represent mean±SEM of 4–11 separate exposures with 3–8 replicates per condition. Statistical significance of rate differences was tested – separately at high and low glucose – by two-way ANOVA with Sidak's post-hoc analysis. ^⁎⁎^ Differs from the equivalent BSA condition (*P* < 0.01).

INS-1E cells exposed for 24 h to palmitate in the presence of high glucose exhibit a MitoSOX oxidation rate that is significantly higher than the rate observed in BSA-exposed control cells (Fig. 1A). This observation agrees with our published results [2] and strongly suggests that palmitate provokes an increase in *mitochondrial* ROS as MitoSOX is a widely used mitochondria-targeted superoxide probe [21]. Not all MitoSOX will accumulate in the mitochondrial matrix, however, and it is thus formally possible that the data in Fig. 1A reflect a palmitate-induced rise in cytoplasmic superoxide that is secondary to stimulated NADPH oxidase activity [22,23]. Importantly, DHE is oxidised at the same rate by palmitate-exposed and BSA control INS-1E cells (Fig. 1B). DHE is the non-targeted equivalent of MitoSOX and its oxidation is therefore dominated by cytoplasmic ROS suggesting that, in our experiments, palmitate has not affected superoxide generation by NADPH oxidase. In a separate set of experiments, we tested the effect of mitochondrial respiratory inhibition with antimycin A on ROS production in INS-1E cells (Fig. 1C). Although the palmitate effect on the MitoSOX oxidation rate was relatively modest in these particular experiments (compare Fig. 1C with Figs. 1A and 3A), it is clear that antimycin A significantly stimulates MitoSOX oxidation in both palmitate-exposed and BSA control cells (Fig. 1C). Antimycin A also increases DHE oxidation a little in BSA control cells, but not to a statistically significant extent. Importantly, antimycin A has no effect on DHE oxidation in palmitate-treated cells (Fig. 1C). Together, the effects of antimycin A support our assertion that glucolipotoxic ROS emerge from mitochondria.

### Palmitate does not affect UCP2 protein

Next, we explored possible UCP2 involvement in the palmitate-induced rise in mitochondrial ROS, in the first instance by probing the effect of palmitate and palmitoleate on UCP2 protein level. Detection and relative quantification of UCP2 protein by Western analysis is complicated by the notorious non-specificity of commercially available UCP2 antibodies. To conclusively identify bands that represent UCP2 in INS-1E samples we routinely include partially purified recombinant human UCP2 protein (hUCP2) and UCP2-depleted cells in our experiments as positive and negative controls, respectively. Fig. 2A shows a typical Western blot that stresses the necessity of these controls: many proteins cross-react with UCP2 antibodies across the entire molecular weight range including a protein doublet at the position to which hUCP2 migrates – only the lower band of this doublet is absent in INS-1E cells transfected with *Ucp2*-targeted siRNA (Fig. 2B). As the intensity of this band consistently falls within the linear range of hUCP2 intensities (Fig. 2C), normalisation to hUCP2 allows relative quantification of UCP2 protein in NEFA-exposed INS-1E cells. Although the absolute values in Fig. 2D bare little relevance given the impurity of hUCP2 and the possible differential antibody reactivity with human and rat proteins, it is clear that the level of UCP2 protein is unchanged in INS-1E cells exposed to palmitate for 24 h at high glucose. Similar exposure to palmitoleate, alone or in combination with palmitate, tends to lower UCP2 protein moderately, but not to a statistically significant extent (Fig. 2D). Equally, palmitate and/or palmitoleate have no obvious effect on UCP2 levels in INS-1E cells transfected with scrambled siRNA (Fig. 2B). The relatively stable UCP2 protein level is consistent with our published observation that mitochondrial proton leak in INS-1E cells is unaffected by NEFA exposure [2].

### UCP2 knockdown amplifies palmitoleate protection against palmitate-induced ROS

Fig. 2B confirms that the NEFA-sensitivity of UCP2 protein is similar in non-transfected cells and cells transfected with scrambled siRNA, and that transfection with *Ucp2*-targeted siRNA leads to UCP2 depletion in both untreated cells and in cells exposed to BSA-conjugated NEFAs or to BSA alone. Therefore, we used this RNA interference approach to directly assess UCP2 involvement in NEFA-induced ROS formation. We measured MitoSOX oxidation in non-transfected and transfected INS-1E cells exposed to palmitate for 24 h at 11 and 4 mM glucose (Fig. 3). In line with data shown in Figs. 1A and C, palmitate exposure causes a significant increase in MitoSOX oxidation in non-transfected cells incubated at high glucose – importantly, the effect of palmitate is near-identical in cells transfected with scrambled siRNA (Fig. 3A). MitoSOX oxidation in UCP2-depleted cells exposed to palmitate tends to be somewhat decreased, but the drop is not statistically significant (Fig. 3A). Palmitate-induced MitoSOX oxidation is relatively modest when cells are exposed at low glucose (Fig. 3B), but again, palmitate-sensitivity does not depend on the presence of UCP2 (Fig. 3B). These data indicate that UCP2 does not mediate palmitate-induced mitochondrial ROS, and does not protect against their formation either.

**Fig. 4:**
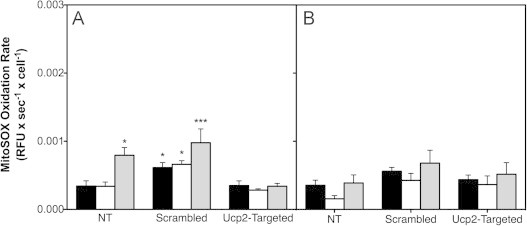
UCP2 knockdown amplifies attenuation by palmitoleate of palmitate-induced mitochondrial ROS. MitoSOX oxidation rates were determined (see Fig. 1) in non-transfected INS-1E cells (NT) or cells transfected with scrambled or *Ucp2*-targeted siRNA oligonucleotides. Cells were exposed for 24 h at 11 or 4 mM glucose (panels A and B, respectively) to BSA-conjugated palmitoleate (white bars), palmitoleate plus palmitate (grey bars) or to BSA alone (black bars). Data represent mean±SEM of 3–5 separate exposures with 3–8 replicates per condition. Statistical significance of rate differences was tested – separately at high and low glucose – by two-way ANOVA with Tukey's post-hoc analysis. ^⁎^, ^⁎⁎⁎^ Differs from the equivalent *Ucp2*-targeted condition (*P*<0.05 and *P*<0.001, respectively).

Irrespective of glucose exposure or the presence of UCP2, MitoSOX oxidation is not affected by palmitoleate (Fig. 4). When administered in combination with palmitate, palmitoleate dampens the rate of palmitate-induced MitoSOX oxidation at high glucose in non-transfected INS-1E cells from 0.002 to 0.0008 RFU s^−1^ cell^−1^ (Figs. 3A and 4 A, respectively), which is consistent with our previously reported data [2]. The preventive effect of palmitoleate is similar in cells transfected with scrambled siRNA (Figs. 3A and 4A). Interestingly, palmitoleate lowers palmitate-induced MitoSOX even further, to about 0.0003 RFU s^−1^ cell^−1^, in UCP2-depleted cells (Fig. 4A). Generally, MitoSOX oxidation in BSA-exposed control cells tends to be marginally higher in cells transfected with scrambled siRNA than in non-transfected or *Ucp2*-transfected cells (Figs. 3 and 4).

### UCP2 knockdown amplifies palmitoleate protection against palmitate-induced cell loss

**Fig. 5:**
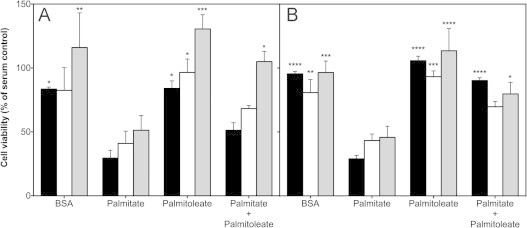
Effect of UCP2 knockdown on the viability of NEFA-exposed INS-1E cells. Viability was determined as described in ‘experimental’ using non-transfected cells (black bars) and cells transfected with scrambled or *Ucp2*-targeted siRNA oligonucleotides (white and grey bars, respectively). Cells were exposed for 24 h at 11 and 4 mM glucose (panels A and B, respectively) to BSA-conjugated palmitate and/or palmitoleate, or to BSA alone. Absolute cell numbers (cf. Fig. 6) were expressed as a percentage of control values obtained with cells grown in standard serum-supplemented growth medium. Values are mean±SEM of 4 independent experiments with 3–5 replicates per treatment. Statistical significance of mean differences was tested – separately at high and low glucose – by two-way ANOVA with Tukey's post-hoc analysis. ^⁎^, ^⁎⁎^, ^⁎⁎⁎^, ^⁎⁎⁎⁎^ Differs from equivalent palmitate condition (*P*<0.05, *P*<0.01, *P*<0.001, *P*<0.0001, respectively).

**Fig. 6:**
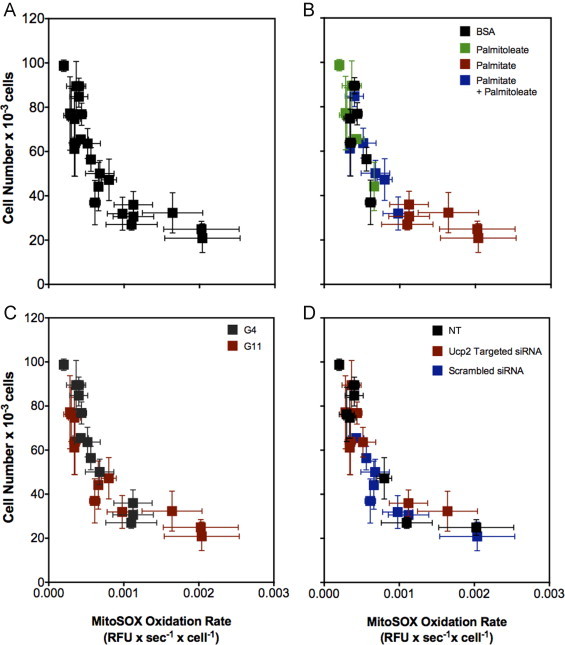
INS-1E cell viability correlates inversely with mitochondrial ROS. Absolute cell number (cf. Fig. 5) was plotted as a function of the MitoSOX oxidation rate (cf. Figs. 3 and 4). Data reflect the behaviour of non-transfected (NT) cells and that of cells transfected with scrambled or *Ucp2*-targeted siRNA oligonucleotides after 24-h exposure at low (G4) and high (G11) glucose to BSA-conjugated palmitate and/or palmitoleate or to BSA alone.

Next, we measured viability of non-transfected and transfected INS-1E cells to probe the effect of UCP2 knockdown on palmitate-provoked cell loss (cf. [2]). Reflecting the effect of transfection on MitoSOX oxidation, absolute numbers of scrambled-transfected BSA control cells are lower than their non-transfected and *Ucp2*-transfected counterparts (see Fig. 6D), but the relative effects of NEFAs on cell viability are comparable between scrambled-transfected and non-transfected cells (Fig. 5). Serum withdrawal tends to have a modest negative effect on the viability of both cell types, particularly at high glucose (Fig. 5A), as reflected by relatively small drops in the number of BSA-exposed control cells. Interestingly, UCP2 knockdown appears to improve cell resistance against the lack of serum, although the viability difference between BSA-exposed cells transfected with scrambled and *Ucp2*-transfected siRNA is not statistically significant (Fig. 5A). At high and low glucose (Figs. 5A and B, respectively), palmitate decreases cell number considerably further, whereas palmitoleate does not exert any effect in addition to serum withdrawal – UCP2 knockdown does not change these NEFA responses. Palmitoleate ameliorates palmitate-induced cell loss, but more strongly at low than high glucose (Fig. 5). Strikingly, the relatively modest protective effect of palmitoleate at high glucose is amplified significantly after UCP2 knockdown (Fig. 5A).

### Inverse correlation between mitochondrial ROS and cell viability

In line with the suggested causative role of ROS in glucolipotoxic beta cell failure [8,9], palmitate affects MitoSOX oxidation and INS-1E cell number in opposite directions (Figs. 3 and 5, respectively). These palmitate phenotypes are both attenuated by palmitoleate – a protection that is regulated by UCP2 (Figs. 4 and 5) – suggesting a mechanistic relation between mitochondrial ROS and INS-1E cell viability. Indeed, combined analysis of the data demonstrates an unequivocal inverse correlation between absolute cell numbers and specific MitoSOX oxidation rates (Fig. 6). The relation between cell number and mitochondrial ROS is not linear as cell loss starts to tail off from a MitoSOX oxidation rate just below 0.001 RFU s^−1^ cell^−1^ (Fig. 6A). A correlative analysis of our data illustrates persuasively that palmitate is indeed toxic to INS-1E cells and that this toxicity is completely prevented by palmitoleate (Fig. 6B). The inverse correlation between cell number and MitoSOX oxidation appears independent of the applied glucose level (Fig. 6C), suggesting that the glucose permissibility on palmitate toxicity is rather weak (Fig. 6B and C). The inverse correlation between cell number and MitoSOX oxidation is the same in non-transfected cells as in cells transfected with scrambled or *Ucp2*-targeted siRNA (Fig. 6D), which suggests that the relatively low number of scrambled-transfected cells is owed to comparably high mitochondrial ROS.

## Discussion

In this paper we show that palmitate-induced ROS in INS-1E cells have a mitochondrial origin (Fig. 1) and that cell viability exhibits a strong inverse correlation with these mitochondrial ROS (Fig. 6). Moreover, we demonstrate that UCP2 is not needed for the palmitate effects on mitochondrial ROS or INS-1E cell viability, but does not ameliorate such effects either (Figs. 3 and 5, respectively). Importantly, we reveal a new and unexpected phenotype as UCP2 appears to attenuate palmitoleate protection against palmitate toxicity (Figs. 4 and 5).

### Palmitate induces mitochondrial ROS

Palmitate increases the oxidation of hydroethidine by INS-1E cells, but only when this superoxide probe is targeted to mitochondria through conjugation to a triphenylphosphonium moiety (Fig. 1A). Palmitate-induced oxidation of the targeted hydroethidine (MitoSOX) is further increased following inhibition of mitochondrial respiration with antimycin A (Fig. 1C). Together, these data indicate that palmitate triggers formation of mitochondrial superoxide in INS-1E cells. A mitochondrial origin of palmitate-induced ROS seems at odds with glucolipotoxicity-provoked expression and activity of a cytoplasmic superoxide-generating NADPH oxidase [24]. Such activity, however, is expected to increase DHE oxidation, which in our experiments appears unaffected by palmitate (Fig. 1B). Superoxide formation by NADPH oxidase is also expected to increase cytoplasmic oxygen uptake – in our hands, palmitate does not change such non-mitochondrial respiration in INS-1E cells [2]. Although the likely nature of palmitate-induced ROS is superoxide, it should be kept in mind that hydroethidine probes are also oxidised, albeit to a lesser extent, by hydrogen peroxide (in the presence of peroxidases) and intracellular oxidoreductases [21]. Additionally, MitoSOX accumulation into the mitochondrial matrix is dependent on mitochondrial membrane potential so changes in MitoSOX oxidation may arise from differential probe availability. As discussed before [13], however, this eventuality is unlikely given the saturating MitoSOX concentration we applied in our experiments. Related, it is conceivable that MitoSOX oxidation is limited by multiple drug resistance (MDR) that could lower net uptake of fluorescent dyes by INS-1E cells. We exposed transfected and non-transfected INS-1E cells to palmitate in the presence of verapamil – an MDR inhibitor [25] – to exclude MDR-related limitations. Under all conditions, verapamil stimulated MitoSOX oxidation modestly, but not to a statistically significant extent (data not shown) – verapamil did not influence NEFA or UCP2 phenotypes.

### Physiological role of UCP2

The first evidence for a beta cell UCP2 emerged some time ago [26] but the physiological role of UCP2 in pancreatic beta cells has still not been established unequivocally [27]. Indeed, the exact molecular role of UCP2 remains subject of debate as this carrier has recently been shown to export carbon compounds from the mitochondrial matrix [28]. Such a function clearly differs from the widely assumed uncoupling activity of UCP2 that would result in partial dissipation of the mitochondrial protonmotive force [29]. In light of the unclear molecular and physiological roles, it is perhaps not surprising that UCP2 involvement in glucolipotoxicity remains contentious [1]. Several functional UCP2 models have been proposed, which include roles in beta cell pathology and consequent development of Type 2 diabetes [15], and in protecting beta cells against oxidative stress [6]. These fundamentally different roles suggest opposite UCP2 involvement in glucolipotoxicity: the pathological model predicts UCP2 is required for palmitate-provoked ROS formation, whereas the protective model predicts UCP2 prevents such formation instead. Our findings do not provide support for either model as UCP2 knockdown does not decrease or increase palmitate-induced mitochondrial ROS production significantly (Fig. 3). Although caution is due when inferring physiological meaning from insulinoma cell data, our results would suggest UCP2 does not mediate harmful effects of palmitate in beta cells and does not protect against such effects either. Consistently, palmitate does not change the level of UCP2 protein (Fig. 2). This lack of effect is discrepant with literature that suggests UCP2 expression is relatively high in palmitate-exposed insulinoma cells as well as in islets from mouse models of Type 2 diabetes and lipotoxicity (reviewed in [30]), and in islets of Type 2 diabetic patients [31]. The discrepancy likely arises from the relative difficulty of quantifying UCP2 protein: (i) measuring mRNA level is insufficient as UCP2 expression is strongly controlled by translation [32] (ii) detecting protein in pancreatic islets is confounded by the presence of UCP2 in both beta and other islet cell types [33] and (iii) Western analysis is complicated by the notorious non-specificity of commercial UCP2 antibodies (Fig. 2A and B). We used positive and negative protein controls to unequivocally identify UCP2 protein in *homogenous* INS-1E cell lysates, and quantified relative protein amount in NEFA-exposed cells by normalising antibody cross-reactivity to partially purified recombinant human UCP2 protein (Fig. 2). Although primary beta cells may respond differently, we are confident that UCP2 protein in INS-1E cells is not affected by overnight palmitate exposure at high glucose.

### UCP2 regulates palmitoleate protection

Palmitoleate protects INS-1E cells against mitochondrial ROS formation and the associated loss of cells (Fig. 6B). Interestingly, UCP2 knockdown amplifies the protective effect of palmitoleate against palmitate-induced ROS (Fig. 4) and cell loss (Fig. 5). These observations suggest an unexpected role for UCP2 in the regulation of beta cell protection by unsaturated NEFAs against cytotoxicity. It has been well established that unsaturated fatty acids protect beta cells against the toxic effects of their saturated counterparts [3] but mechanistic understanding of this phenomenon is incomplete [34]. Interestingly, cytoprotection is not restricted to lipotoxic stress as unsaturated NEFAs also prevent cell loss after serum withdrawal or cytokine exposure [35]. In this respect, it is worth notice that UCP2 knockdown protects against the moderate viability loss of control INS-1E cells that were deprived from serum and exposed overnight to BSA alone at high glucose (Fig. 5A). Although our data do not explain how UCP2 activity may dampen palmitoleate protection, they firmly implicate mitochondrial energy metabolism in this poorly understood process.

### Glucose dependence of palmitate toxicity

NEFA-induced beta cell defects are often – but not always [35] – exclusively observed at a high glucose level, which is why such defects are generally referred to as glucolipotoxicity [36]. Indeed, our previously published experiments showed statistically insignificant effects of palmitate on MitoSOX oxidation and INS-1E cell viability when administered overnight at low glucose [2]. Although the effect on MitoSOX oxidation remains insignificant in the current experiments, palmitate consistently tends to increase mitochondrial ROS in non-transfected and transfected cells following exposure at low glucose (Fig. 3B), albeit to a lesser extent than at high glucose (Fig. 3A). Consistently, palmitate causes significant cell loss in non-transfected and transfected cells at both high and low glucose (Fig. 5). Quantitative differences with our previously reported cell numbers [2] likely arise from application of different cell viability assays, but the glucose dependence of the palmitate viability phenotype appears weak irrespective of such experimental differences. Indeed, our combined analysis of mitochondrial ROS and INS-1E cell viability data confirms that palmitate is most toxic at high glucose, but causes significant damage at low glucose too (Figs. 6B and C).

### Final remarks

In conclusion, our results support the notion that oxidative stress contributes to pancreatic beta cell glucolipotoxicity and shed important new light on the elusive mechanism by which unsaturated NEFAs protect against the harmful effects of their saturated counterparts. In addition, our findings inform ongoing debate on the physiological role of the beta cell UCP2.
